# Passive proton therapy vs. IMRT planning study with focal boost for prostate cancer

**DOI:** 10.1186/s13014-015-0522-5

**Published:** 2015-10-24

**Authors:** Inhwan Yeo, Prashanth Nookala, Ian Gordon, Reinhard Schulte, Stanley Barnes, Abiel Ghebremedhin, Ning Wang, Gary Yang, Ted Ling, David Bush, Jerry Slater, Baldev Patyal

**Affiliations:** Department of Radiation Medicine, Loma Linda University Medical Center, 11234 Anderson St., Loma Linda, CA 92354 USA

**Keywords:** Intraprostatic boost, Proton plan, IMRT plan

## Abstract

**Background:**

Exploiting biologic imaging, studies have been performed to boost dose to gross intraprostatic tumor volumes (GTV) while reducing dose elsewhere in the prostate. Interest in proton beams has increased due to superior normal-tissue sparing they afford. Our goal was to dosimetrically compare 3D conformal proton boost plans with intensity-modulated radiation therapy (IMRT) plans with respect to target coverage and avoiding organs at risk.

**Methods:**

Treatment planning computer tomography scans of ten patients were selected. For each patient, two hypothetical but realistic GTVs each with a fixed volume were contoured in different anatomical locations of the prostate. IMRT and proton beam plans were created with a prescribed dose of 50.4 Gy to the initial planning target volume (PTV) including the PTV of the seminal vesicles (PSV), 70.2 Gy to the PTV of the prostate (PPS), and 90 Gy to the PTV of the gross tumor volumes (PGTVs). For proton plans, uncertainties of range and patient setup were accounted for; apertures were adjusted until the dose-volume coverage of PTVs matched that of the IMRT plan. For both plans, prescribed PTV doses were made identical to allow for comparing normal-tissue doses.

**Results:**

Protons delivered more homogeneous but less conformal doses to PGTVs than IMRT did and comparable doses to PSV and PPS. Volumes of bladder and rectum receiving doses higher than 65 Gy were similar for both plans. However, volumes receiving less than 65 Gy were significantly reduced, i.e., protons reduced integral dose by 45.6 % and 26.5 % for rectum and bladder, respectively. This volume-sparing was also seen in femoral heads and penile bulb.

**Conclusions:**

Protons delivered comparable doses to targets in dose homogeneity and conformity and spared normal tissues from intermediate-to-low doses better than IMRT did. Further improvement of dose sparing and changes in homogeneity and conformity may be achieved by reducing proton range uncertainties and from implementing intensity modulation.

## Background

There has been a shift of radiation treatment paradigms towards organ-sparing focal treatments of the biologically significant lesions in advanced prostate cancer cases. This approach was envisioned by Ling et al. [1] who predicted that future radiation therapy would be based on conforming high doses to lesions of significant cancer, identified with biological imaging, while delivering lower doses to surrounding tissues.

This approach has been explored in planning studies of prostate cancer with intra-prostatic legions with intensity-modulated radiation therapy (IMRT) [2–6] as well as Phase I-II clinical studies [7–10]. The former has reported increased therapeutic ratios due to the local dose escalation and the latter reported comparable toxicity to treatments without local dose escalation [8–10]. The focal boost idea was extended to the use of proton beams by Schulte and Li [11].

External beam therapy with protons rather than photons is a topic of active discussion. At present, only a few comparative studies between IMRT and passively modulated proton therapy have been performed for prostate cancer. It was found that without compromising prostate coverage better sparing of normal tissues may be possible with proton beams [12, 13]. In this study, we comparatively evaluated boost treatment plans using passive proton beams and X-ray IMRT in ten prostate cancer cases by placing two hypothetical but realistic index GTVs into their prostate. The objective was to compare proton and IMRT plans with respect to target coverage and avoidance of normal tissues. To our knowledge, this is the first comparison of this kind.

## Methods

### Patient selection and volume definition

**Fig. 1:**
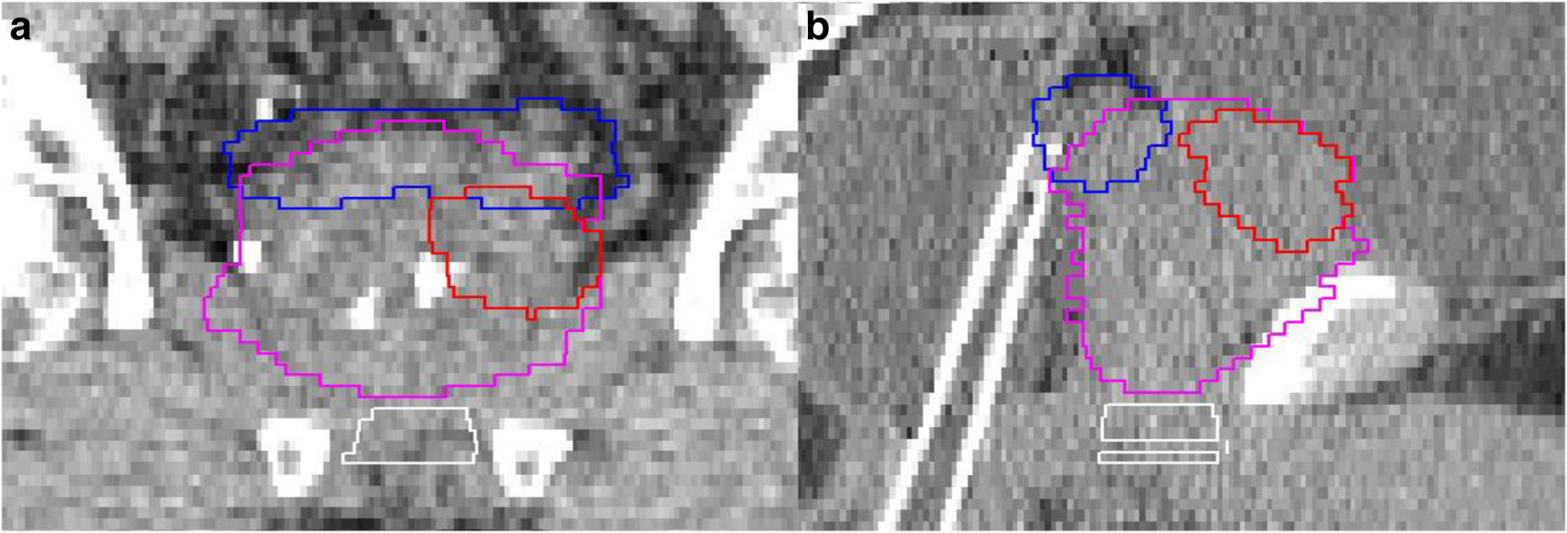
Coronal (**a**) and sagittal (**b**) images with GTVs expanded with a planning margin. The prostate is in purple; the GTV is in red; the seminal vesicle is in blue

Ten computer tomography images, extending from L4 to 2 cm below the ischium, were selected from patients with intermediate- and high-risk prostate cancer treated by passive proton beams at Loma Linda University Medical Center. The study was approved by the Institutional Review Board at our institution. The prostate (PS) was delineated and seminal vesicles were contoured bilaterally and up to 1 cm proximally to the prostate. These regions formed the clinical target volumes (CTVs). Within PS, we chose two spherical index lesion GTVs with a volume of approximately 2 cc as shown in Fig. 1; one lesion was placed in the left lateral peripheral prostate zone, the most common location for macroscopic prostate cancer [2, 5], and centered in superior-inferior direction; the other was placed in the sagittal midplane adjacent to the bladder. The selection of the two GTVs was based on the following considerations: more than two lesions and/or a larger size would have excluded the use of a local boost; the size of 2 cc is representative of index lesions [2, 5]; the second GTV near the bladder, although not a common location, creates a dosimetric challenge to show a potential advantage of one technique over the other. The GTVs were delineated, giving them a spherical shape with each volume of 2 cc within +/− 10 %.

Bladder, bladder wall, rectum, and rectal wall were also delineated. The rectum and its wall were delineated from the level of the ischial tuborisities to the recto-sigmoid flexure. Right and left femoral heads, penile bulb, and external body were contoured as well. Patients were immobilized in a low-density foam shell placed inside a half-cylinder plastic pod. A setup-uncertainty/internal motion margin (SM) of 5 mm was added to the CTVs and GTVs according to our institutional policy, based on the use of rectal balloons [14]. This resulted in the planning target volumes for the prostate (PPS), the seminal vesicles (PSV), and the GTVs (PGTVs). Both proton and IMRT plans were generated on the patients only excluding the pod in this planning study.

### Dose prescription

In our practice, we deliver 50.4 Gy to the PSV and 81 Gy to the PPS. In this study, we boosted the PGTVs to a dose of 90 Gy, while reducing the dose to the rest of PPS with microscopic disease to 70.2 Gy. We maintained 50.4 Gy to the PSV. Three sessions of 28, 11, and 11 fractions at 1.8 Gy/fraction can be delivered. As CTVs were expanded by the SM, some of the resulting PTVs overlapped with each other. Therefore, the PTVs effectively included PGTVs, PPS subtracted by PGTVs (PPS-PGTVs), and PSV-PPS.

### IMRT planning

Planning was performed with 7 equally spaced 6 MV beams starting from a gantry angle of 0° using a Varian iX accelerator (Varian Medical Systems, Inc., Palo Alto, CA) with a leaf width of 0.5 cm. The planning was done on the Odyssey planning system (Optivus Proton Therapy, Inc., CA) with a voxel size of 0.3 x 0.3 x 0.25 cm^3^. For inverse optimization, we imposed constraints of the Radiation Therapy Oncology Group (RTOG) Protocol 0815 as follows [15]: all target volumes should receive their prescribed doses (PDs) to at least 98 % of their volumes; the minimum dose (*D*_*min*_) within each target should be greater than 95 % of each PD; the maximum dose (*D*_*max*_) within each target should not exceed 110 % of each PD (variation). Bladder constraints are as follows: no more than 15 % volume receives a dose that exceeds 80 Gy (V80 < 15 %); V75 < 25 %; V70 < 35 %; V65 < 50 %. The rectum constraints are: V75 < 15 %; V70 < 25 %; V65 < 35 %; V60 < 50 %. The mean penile bulb dose was constrained to < 52.5 Gy. In addition to the RTOG constraints, the maximum dose to femoral heads was limited to 50 Gy. The optimization was repeated until the constraints were met and no significant improvement in tumor coverage and organ savings was made by further optimization.

### Proton planning

Two parallel-opposing lateral beams were applied to the combination of PPS and PSV to deliver a dose of 50.4 Gy, and a second set of two reduced beams was applied to the PPS to a total of 70.2 Gy. Finally, a left-lateral beam was applied to boost the dose to the two GTVs to a total of 90 Gy. Lateral beams have been traditionally used in proton therapy because they are most robust with respect to range uncertainties and mostly exclude bladder and rectum with their distal edges not pointed toward them [14, 16].

Appropriate distal and proximal margins were added to ensure coverage of the CTVs by the spread-out proton Bragg peak (SOBP) of each beam. For the margin determination, we employed a range uncertainty of 0.3 cm for uncertainties in accelerator energy, beam scattering foil thickness, and compensator bolus [17]. We used an additional uncertainty of 3.5 % for CT accuracy (CT number conversion to proton stopping power), as shown in Eqs. (1) and (2).

1 $$ \mathrm{Distal}\ \mathrm{margin} = 0.035 \bullet \mathrm{distal}\ \mathrm{C}\mathrm{T}\mathrm{V}\ \mathrm{depth} + 0.3\ \mathrm{cm} $$

2 $$ \mathrm{Proximal}\ \mathrm{margin} = 0.035 \bullet \mathrm{proximal}\ \mathrm{C}\mathrm{T}\mathrm{V}\ \mathrm{depth} + 0.3\ \mathrm{cm} $$

Perpendicular to the beam direction, we adjusted the aperture margins until PTVs were dosimetrically covered, meeting the above target coverage requirements. This was done to compare the proton plans with the IMRT plans, which utilized the PTV concept. Of note, this approach was also adopted by the Children’s Oncology Group Protocol ACNS0831 when allowing proton therapy and IMRT [18].

To account for setup uncertainty that can affect proton range and to ensure full lateral scattering [19], the compensator (bolus) for each beam was “smeared” perpendicular to the beam direction by applying a smearing radius given by Eq. (3)

3 $$ \mathrm{Smearing}\ \mathrm{radius} = {\left\{\mathrm{S}{\mathrm{M}}^2 + {\left[0.03 \bullet \mathrm{bolus}\ \mathrm{thickness}\ \mathrm{of}\ \mathrm{range}\ \mathrm{shift}\right]}^2\right\}}^{0.5}. $$

### Plan evaluation

Plans were evaluated in terms of meeting the imposed planning constraints, integral dose (Gy cm^3^), and the inhomogeneity coefficient (IC), defined for each PTV as

4 $$ IC=\frac{D_{\max }-{D}_{\min }}{D_{mean}}, $$

where *D*_*max*_, *D*_*min*_, and *D*_*mean*_ are the maximum, minimum, and mean PTV dose, respectively. The highest possible *IC* is zero. In addition, a conformity number (*CN*) as described in eq. (5) was used [20], which addresses both target coverage and normal tissue avoidance.

5 $$ CN=\left[\frac{PTV\  encompassed\ by\ 95\%\  isodose}{PTV}\right]\times \left[\frac{PTV\  encompassed\ by\ 95\%\  isodose}{95\%\  isodose\  volume}\right] $$

The highest possible *CN* is one. For comparative dose evaluation of IMRT and proton plans with respect to normal tissues, we applied the same dose prescription to both modalities. The dosimetric data and evaluation parameters were averaged over the ten cases and statistically evaluated by the Mann-Whitney test.

## Results and discussion

**Fig. 2:**
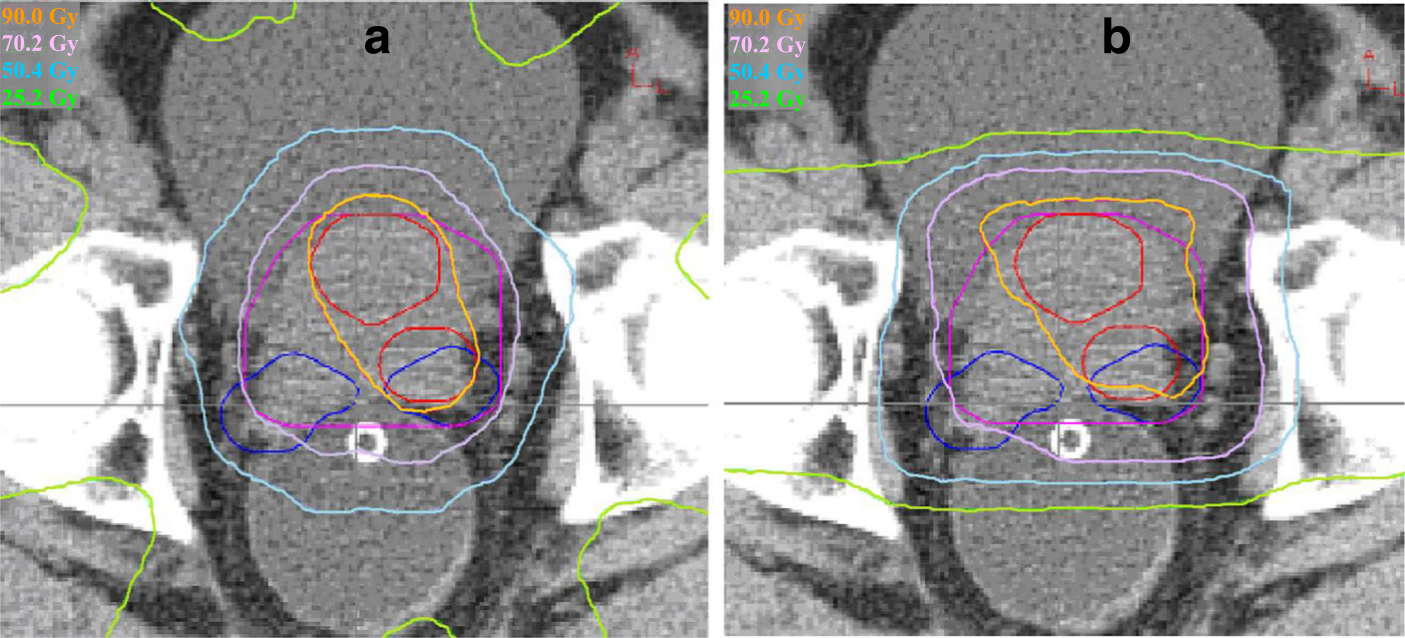
IMRT (**a**) and proton (**b**) plans. PGTV(red), PPS(purple), and PSV(blue) are enclosed by various isodose lines

Dose distributions of IMRT and proton plans are shown in Fig. 2. The dose distribution of the IMRT plan was contributed by conformal beam arrangement, while that for the proton plan by lateral arrangement.

**Fig. 3:**
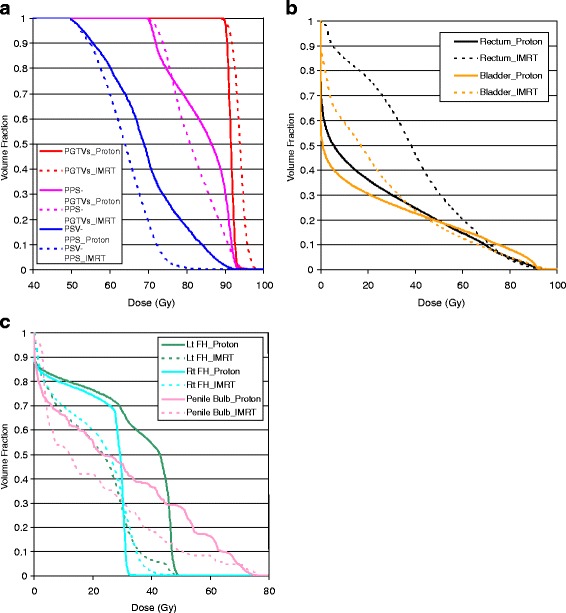
IMRT and proton plan comparison in dose volume histogram. **a** DVH for planning target volumes; **b** DVH for rectum and bladder; **c** DVH for femoral heads and penile bulb

Dose volume histograms of the two plans are provided in Fig. 3. The averaged planning data and plan evaluation parameters for the PTVs and normal tissues are given in Tables 1, 2, and 3. Table 1 shows that for both proton and IMRT plans, each PTV received a *D*_*min*_ greater than 95 % of the PD. For brevity, the two PGTVs were evaluated together. For both plans, *D*_*max*_ for the PGTVs was smaller than 110 % of the PD. However, the maximum dose constraints for PSV-PPS and PPS-PGTVs were exceeded due to their proximity to the PGTVs.

**Table 1:** Average dosimetric data and plan evaluation parameters for the PTVs

Target	Plans	Prescription	Inhomogeneity						Conformity	
		Volume^a^	*D*_*min*_^b^	*D*_*max*_^b^	*D*_*mean*_^c^	p*	*IC*	p*	*CN*	p*
PSV-PPS	Proton	99.0 %	96.3 %	175.1 %	69.3	**<0.001**	0.571	**0.029**	0.051	0.796
	IMRT		96.6 %	158.7 %	63.7		0.491		0.054	
PPS-PGTVs	Proton	99.2 %	98.4 %	133.1 %	83.9	**0.019**	0.290	**<0.001**	0.328	0.143
	IMRT		96.2 %	135.0 %	81.7		0.333		0.392	
PGTVs	Proton	98.7 %	99.3 %	104.0 %	91.6	**<0.001**	0.046	**<0.001**	0.216	**<0.001**
	IMRT		97.8 %	107.9 %	93.9		0.097		0.358	

*p-value associated with the values to the left; significant values are bold

^a^Minimum volume covered by the PD

^b^Relative to PD (%); defined to 0.02 cc

^c^in Gy

**Table 2:** Average dosimetric data and plan evaluation parameters for rectum and bladder

		V90	p*	V75< 15 %^b^	p*	V70< 25 %^b^	p*	V65< 35 %^b^	p*	V60< 50 %^b^	p*	*D*_*mean*_^a^	p*	*ID* ^c^	p*	*ID* ^c^ (w)	p*
Rectum (w = wall)	Proton	1.1 %	0.315	6.7 %	0.631	9.7 %	0.315	12.3 %	**0.029**	14.6 %	**0.009**	21.5	**<0.001**	50.1	**<0.001**	15.2	**<0.001**
	IMRT	0.7 %		7.3 %		11.4 %		15.7 %		20.4 %		38.9		92.1		25.7	
Bladder (w = wall)		V90	p*	V80< 15 %^b^	p*	V75< 25 %^b^	p*	V70< 35 %^b^	p*	V65< 50 %^b^	p*	*D*_*mean*_^a^	p*	ID ^c^	p*	ID ^c^ (w)	p*
	Proton	2.8 %	0.089	8.1 %	**0.035**	10.0 %	0.075	12.1 %	0.165	14.1 %	0.218	19.7	0.190	60.6	**0.043**	21.1	0.052
	IMRT	1.7 %		5.0 %		6.7 %		8.6 %		10.5 %		24.5		82.9		25.7	

*p-value associated with the values to the left; significant values are bold

^a^in Gy

^b^RTOG0815 constraint

^c^Integral dose in Gy cm^3^

**Table 3:** Average dosimetric data and plan evaluation parameters for other normal tissues

		*D*_*mean*_^a^	p*	*ID* ^b^	p*
Rt Femoral Head	Proton	23.0	0.105	35.3	0.529
	IMRT	20.7		31.4	
Lt Femoral Head	Proton	32.7	**<0.001**	49.5	**0.002**
	IMRT	19.4		29.5	
Penile Bulb	Proton	28.6	0.529	1.3	0.481
	IMRT	20.7		0.9	
Normal Tissues outside PTVs	Proton	4.8	**0.002**	771.6	**0.001**
	IMRT	7.3		1209.8	

*p-value associated with the values to the left; significant values are bold

^a^in Gy

^b^Integral dose in Gy cm^3^

The average inhomogeneity indices of PPS-PGTVs and PGTVs was larger for IMRT plans (33.3 % and 9.7 %, respectively) compared to the proton plans (29 % and 4.6 %). This characteristic, i.e., the high-dose tail, is common to IMRT plans, whereas proton plans provide more homogeneous dose due to SOBP (Fig. 3a). However, for the PSV-PPS, the proton plan was more inhomogeneous than the IMRT plan (57.1 % vs. 49.1 %) due to spill over of higher doses from the PGTV coverage.

The average conformity indices were higher for the IMRT plans, although the difference was only significant for the PGTVs (35.8 % vs. 21.6 %). This can be explained by the fact that proton plans employed two lateral beam directions, whereas the IMRT plans were intensity-modulated with 7 directions.

The rectal volumes receiving doses >65 Gy were similar for IMRT and proton plans; the volumes receiving doses ≤65 Gy were reduced for protons, leading to an integral dose (*ID*) to the rectum reduced by 45.6 %, and to the rectal wall by 40.9 % (Table 2; Fig. 3b). The differences between protons and IMRT were somewhat similar for bladder; the volume receiving doses ≤45 Gy was smaller for proton beams (Table 2;Fig. 3b), leading to an *ID* to the bladder reduced by 26.5 %, and to the bladder wall by 17.9 %. Doses delivered to left femoral heads were significantly higher for the proton plans (Table 3;Fig. 3c), owing to the left lateral proton beam boosting the PGTV. The right lateral femoral head and penile bulb received similar doses for proton and IMRT plans. As seen in the table, all plans met the RTOG dose constraints. Concerning normal tissues outside the PTVs, proton plans delivered an *ID* that was 36.3 % less than that of IMRT plans.

**Fig. 4:**
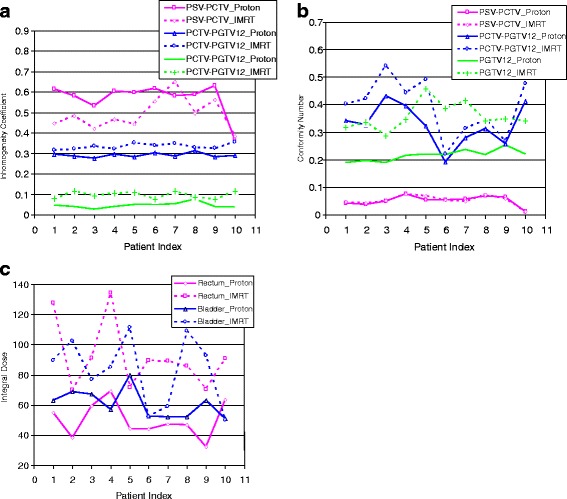
Inhomogeneity coefficients (**a**), conformity numbers (**b**), and integral dose (**c**) for PTVs of ten patients

The individual plan data and parameters for ten patients are provided in Fig. 4 and the appendix. Figure 4 shows the above trend, found as an average over 10 patients, individually.

Trofimov et al. [13] and Vargas et al. [12] compared IMRT and passive proton plans for prostate cancer. Different from our study, these investigators did not use a focal GTV boost; Trofimov et al., in addition to opposing lateral beams, adopted oblique beam angles and one IMPT plan; Vargas et al. optimized beam angles and reduced standard aperture margins by up to 30 % to spare bladder and rectum. Therefore, our findings of normal tissue coverage are not directly comparable to those by Trofimov and Vargas et al., although in agreement with them, substantially smaller dose was received by the volumes of rectum and bladder exposed to low-to-intermediate doses for proton plans.

Proton beams deliver more conformal and homogeneous dose, thanks to proximal and distal dose fall off and SOBP, than photons do. In this study, this disadvantage of photon beams was compensated by employing greater beam numbers and intensity modulation. Even without intensity modulation, passively scattered proton beams, using a lateral orientation, demonstrated dosimetric advantages in the low-to-intermediate dose range for critical organs and competing dose homogeneity and conformity for PTVs. Adoption of different orientations and/or increased beam number would further improve the proton plans (i.e. conformity), but we chose not to do so here, as GTVs are within CTV, another target, and the lateral beams are the most “robust” toward range uncertainties [14, 16].

Range uncertainties may decrease, and the conformity to PGTVs may change if we use scanning beams and improve imaging techniques [21, 22]. Additional range uncertainty due to uncertainty in RBE [23] may also be taken into account by implementing biologically-weighted treatment planning. Utilization of intensity modulation may also alter the conformity and the homogeneity to PGTVs and the homogeneity outside the PGTVs and inside PCTVs (PPS in this study), while the advantages in the low-do-intermediate dose and integral dose are retained. Lastly, as intensity modulation is implemented, there could be an interplay between interfraction organ motion and IMPT dose delivery that should be studied using repeated CT studies, deformable image registration, and dose summation [16].

For successful boost therapy, enhanced dose conformity to the PGTVs and homogeneity outside the PGTV would be better accepted by the treating physician. How much this will be translated into improved outcomes can only be demonstrated in future clinical trials. The boosting concept for intraprostatic dominant lesions is a rather new idea that has yet to be tested in (best randomized) clinical trials stratified by boosting technique. Initial single-institution experiences cited in the introduction of our paper, point toward feasibility and a favorable clinical outcome, but further clinical evidence needs to be accumulated and should also include novel modalities such as protons.

This paper intended to perform a dosimetric comparison with existing and widely practiced proton beam delivery techniques. In the future, one could also consider an integrated simultaneous boost technique rather than sequential delivery of the GTV boost. A simultaneous boost would be potentially more accurate because it would not require a different patient setup. The implementation of this technique can be made possible by IMPT.

This study reported reduction of volumes receiving low-to-intermediate dose and integral dose by proton beams. The use of protons for reducing clinical toxicity is a very active area of ongoing clinical study [24]. and currently the most convincing data that a reduction of volume receiving low-to-intermediate dose afforded by proton therapy to less side effects came from the experience in head and neck cancer [25]. Regarding the impact of lower integral doses, the clinical benefits are also demonstrated in gastrointestinal radiotherapy, e.g., [26, 27]. We believe that benefits will eventually be seen when more normal tissue is spared with proton radiotherapy of prostate cancer, but this needs further evidence from prospective clinical trials.

## Conclusions

We have performed a first study that compares proton plans with IMRT for prostate cancers with intraprostatic lesions. Compared with IMRT, passive proton beams produced comparable doses to targets in dose homogeneity and conformity and spared normal tissues from intermediate-to-low doses better. Therefore, passive proton beams can be an advantageous choice for treating prostate cancers with a local boost. Further improvement of dose sparing and changes in homogeneity and conformity may be achieved by reducing proton range uncertainties and from implementing intensity modulation.

## Appendix

**Table 4:** Planned dose in targets

		PSV-PCTV						PCTV-PGTVs						PGTVs					
Patient	Plan	Prescription	Inhomogeneity				Conformity	Prescription	Inhomogeneity				Conformity	Prescription	Inhomogeneity				Conformity
		Volume^a^	*D*_*min*_^b^	*D*_*max*_^b^	*D*_*mean*_^c^	*IC*	*CN*	Volume	*D*_*min*_^b^	*D*_*max*_^b^	*D*_*mean*_^c^	*IC*	*CN*	Volume^a^	*D*_*min*_^b^	*D*_*max*_^b^	*D*_*mean*_^c^	*IC*	*CN*
1	Proton	99.1 %	97.2 %	180.2 %	68.1	0.614	0.043	99.3 %	97.7 %	132.9 %	83.5	0.296	0.342	98.9 %	99.2 %	103.8 %	91.9	0.045	0.189
	IMRT	99.1 %	95.6 %	152.2 %	63.6	0.448	0.048	99.3 %	96.2 %	133.0 %	82.1	0.316	0.404	98.9 %	98.8 %	107.0 %	93.3	0.079	0.319
2	Proton	99.3 %	99.0 %	180.0 %	70.1	0.582	0.039	98.8 %	98.3 %	132.9 %	85.1	0.285	0.328	98.4 %	99.7 %	103.8 %	91.2	0.041	0.197
	IMRT	99.3 %	96.6 %	158.1 %	64.1	0.484	0.043	98.8 %	96.2 %	134.0 %	82.7	0.322	0.422	98.4 %	96.3 %	108.2 %	93.5	0.114	0.336
3	Proton	98.5 %	95.0 %	165.7 %	66.8	0.533	0.049	98.5 %	99.3 %	131.1 %	80.7	0.276	0.431	98.2 %	99.4 %	102.2 %	91.1	0.027	0.189
	IMRT	98.5 %	95.6 %	148.4 %	62.2	0.428	0.052	98.5 %	95.3 %	133.9 %	81.0	0.334	0.542	98.2 %	96.8 %	106.2 %	93.3	0.091	0.286
4	Proton	99.4 %	97.8 %	178.2 %	67.0	0.604	0.076	99.3 %	97.2 %	132.3 %	84.1	0.294	0.397	98.8 %	99.2 %	103.3 %	91.2	0.041	0.215
	IMRT	99.4 %	98.0 %	155.6 %	62.4	0.465	0.076	99.3 %	95.7 %	133.6 %	82.9	0.321	0.443	98.8 %	97.3 %	108.4 %	93.9	0.106	0.346
5	Proton	99.4 %	95.4 %	181.7 %	72.9	0.597	0.054	99.1 %	98.6 %	132.3 %	84.5	0.281	0.323	98.7 %	98.9 %	103.9 %	91.4	0.049	0.221
	IMRT	99.4 %	96.8 %	152.6 %	64.9	0.433	0.069	99.1 %	95.0 %	135.5 %	81.0	0.350	0.491	98.7 %	97.3 %	108.8 %	94.5	0.109	0.459
6	Proton	98.6 %	96.0 %	183.5 %	71.3	0.619	0.054	99.2 %	98.3 %	134.2 %	83.5	0.302	0.193	99.3 %	99.7 %	104.8 %	92.2	0.050	0.219
	IMRT	98.6 %	95.2 %	166.9 %	65.1	0.554	0.055	99.2 %	96.3 %	134.9 %	80.4	0.337	0.220	99.3 %	98.9 %	106.6 %	93.1	0.074	0.386
7	Proton	98.9 %	98.2 %	181.9 %	72.7	0.581	0.056	99.6 %	99.3 %	134.2 %	85.7	0.286	0.281	98.5 %	98.4 %	103.9 %	92.0	0.053	0.237
	IMRT	98.9 %	95.2 %	177.2 %	63.4	0.652	0.051	99.6 %	96.9 %	137.0 %	81.4	0.347	0.313	98.5 %	97.9 %	110.0 %	95.3	0.114	0.415
8	Proton	98.8 %	95.4 %	172.0 %	65.6	0.589	0.069	99.3 %	98.1 %	135.9 %	84.8	0.313	0.313	99.0 %	99.4 %	107.3 %	92.0	0.077	0.218
	IMRT	98.8 %	96.4 %	158.5 %	63.1	0.496	0.074	99.3 %	96.6 %	134.5 %	81.0	0.328	0.346	99.0 %	99.1 %	108.2 %	94.0	0.087	0.341
9	Proton	98.2 %	92.5 %	183.7 %	72.8	0.632	0.063	99.5 %	97.9 %	132.6 %	86.6	0.282	0.259	99.3 %	99.4 %	103.3 %	91.9	0.038	0.255
	IMRT	98.2 %	97.2 %	168.8 %	64.2	0.562	0.060	99.5 %	98.0 %	136.8 %	83.5	0.326	0.268	99.3 %	99.0 %	106.8 %	93.7	0.075	0.349
10	Proton	99.3 %	96.4 %	143.7 %	65.5	0.363	0.012	99.6 %	99.0 %	132.2 %	80.4	0.290	0.414	98.0 %	99.6 %	103.3 %	91.3	0.037	0.221
	IMRT	99.3 %	99.2 %	148.8 %	64.9	0.385	0.012	99.6 %	95.9 %	136.5 %	80.6	0.354	0.477	98.0 %	96.6 %	108.7 %	94.2	0.116	0.341

^a^Minimum volume to which prescribed dose is planned

^b^Relative to the prescribed dose in percent

^c^In Gy

**Table 5:** Planned dose in rectum and bladder

		Rectum									Bladder								
Patient	Plans	V90	V75	V70	V65	V60	V40	*D*_*mean*_^a^	*ID* ^b^	*ID* ^b^ (w^c^)	V90	V80	V75	V70	V65	V40	*D*_*mean*_^a^	*ID* ^b^	*ID* ^b^ (w^c^)
1	Proton	0.5	4.4	6.9	9.1	10.8	17.4	15.3	54.8	18.2	1.0	3.6	4.4	5.4	6.5	11.8	10.4	63.0	23.5
	IMRT	0.2	6.8	10.1	13.4	16.8	38.9	35.6	127.8	35.5	0.5	2.4	3.2	4.2	5.3	14.5	14.8	89.5	29.3
2	Proton	1.3	7.1	8.6	10.3	12.1	22.2	19.6	38.2	11.3	2.3	6.2	7.5	8.7	9.8	16.1	14.3	69.0	23.9
	IMRT	0.5	7.1	9.9	13.1	16.8	41.0	36.0	70.1	20.9	1.7	4.2	5.4	6.7	8.1	18.8	21.2	102.6	30.7
3	Proton	0.5	6.7	11.4	15.4	18.5	30.9	25.4	59.6	17.9	2.9	7.8	10.2	12.7	15.1	23.2	19.9	67.1	21.5
	IMRT	0.5	6.0	9.9	13.9	18.3	43.9	38.9	91.2	26.0	1.6	4.8	6.7	8.8	10.8	23.3	22.8	77.0	23.5
4	Proton	1.7	7.9	10.2	12.3	14.3	25.3	21.7	69.4	18.9	2.6	7.3	9.1	10.7	12.1	19.8	17.5	57.1	20.2
	IMRT	1.3	10.1	13.9	18.2	23.2	54.5	41.9	134.3	30.7	1.6	4.7	6.3	7.9	9.6	24.5	26.1	85.1	28.2
5	Proton	0.6	7.4	10.3	13.5	15.8	26.1	22.9	44.6	16.3	2.5	7.3	9.0	10.5	12.0	18.6	16.5	79.6	31.7
	IMRT	0.5	4.9	8.2	11.7	15.7	40.1	36.8	71.7	23.1	0.8	2.5	3.8	5.2	6.7	20.4	23.1	111.6	35.8
6	Proton	0.0	2.7	4.1	5.9	8.0	19.4	17.4	44.2	11.7	2.6	8.9	11.3	13.9	16.8	28.0	24.2	52.4	17.1
	IMRT	0.0	4.5	10.1	15.8	20.6	45.2	35.2	89.4	22.5	1.2	6.2	9.0	11.6	14.1	25.7	24.2	52.3	16.7
7	Proton	2.6	7.9	10.0	11.4	13.9	24.2	21.3	47.3	15.4	3.4	11.3	14.7	17.5	20.2	34.8	30.4	52.0	19.1
	IMRT	1.5	9.5	13.7	18.6	24.6	51.2	40.3	89.3	28.2	1.9	5.0	6.9	9.0	11.6	31.3	34.6	59.1	22.7
8	Proton	2.2	9.2	11.4	13.6	15.9	25.3	22.0	46.8	13.1	0.7	2.6	3.3	4.1	4.9	8.7	7.7	52.0	16.1
	IMRT	0.8	6.8	10.8	15.9	21.6	48.0	40.4	85.9	23.1	0.7	2.6	3.5	4.5	5.6	14.8	16.1	108.9	25.9
9	Proton	0.3	5.2	6.8	8.3	10.0	18.4	18.1	32.2	11.1	2.3	6.5	8.1	9.6	11.1	17.9	15.8	63.1	18.8
	IMRT	0.5	7.9	11.8	15.9	20.3	49.3	39.4	70.3	20.9	2.7	6.4	7.9	9.3	10.8	22.7	23.2	92.7	24.0
10	Proton	1.3	8.4	17.3	23.0	26.9	38.1	31.0	63.4	18.0	7.8	19.2	22.6	28.2	32.3	47.2	40.0	51.0	18.8
	IMRT	1.0	9.3	15.7	20.8	25.7	53.1	44.3	90.8	26.3	4.3	10.8	14.7	18.6	22.5	41.8	39.4	50.2	20.2

Volumes are provided in percent

^a^In Gy

^b^Integral dose in Gycm^3^

^c^w implies rectal or bladder wall

**Table 6:** Planned dose in other normal tissues

		Rt FH			Lt FH			PB		Ext-PCTV	
Patient	Plans	*D*_*max*_^a^	*D*_*mean*_^a^	*ID* ^b^	*D*_*max*_^a^	*D*_*mean*_^a^	*ID* ^b^	*D*_*mean*_^a^	*ID* ^b^	*D*_*mean*_^a^	*ID* ^b^
1	Proton	32.1	29.9	20.8	50.0	41.4	28.9	64.7	1.6	3.4	838.2
	IMRT	41.1	31.2	21.7	35.4	25.2	17.6	56.6	1.4	5.9	1479.3
2	Proton	30.7	23.4	30.7	48.1	34.0	43.2	43.9	1.8	3.8	325.6
	IMRT	39.6	15.1	19.8	43.0	12.9	16.3	27.0	1.1	5.8	497.4
3	Proton	32.9	20.4	37.0	50.2	29.3	50.8	34.0	0.9	5.8	992.8
	IMRT	47.4	23.4	42.4	54.5	20.8	35.9	17.7	0.5	8.5	1445.7
4	Proton	32.9	24.3	49.1	49.0	33.6	69.8	11.2	0.9	4.5	952.6
	IMRT	42.1	18.9	38.1	43.4	18.4	38.1	5.8	0.5	7.1	1511.3
5	Proton	31.6	20.9	27.4	47.9	27.9	35.4	40.3	1.6	8.9	757.3
	IMRT	35.9	16.7	21.9	43.8	14.3	18.2	22.3	0.9	12.1	1033.3
6	Proton	32.5	24.3	44.6	49.2	35.5	63.9	6.6	0.4	4.5	745.6
	IMRT	44.8	21.3	39.1	45.2	21.3	38.4	4.1	0.3	6.6	1092.7
7	Proton	32.9	24.1	45.1	49.6	33.2	59.2	27.8	2.1	3.7	786.8
	IMRT	41.9	20.5	38.3	42.0	17.4	31.1	20.9	1.6	6.0	1298.5
8	Proton	39.7	20.5	32.6	51.7	31.7	50.3	0.6	0.0	3.7	744.5
	IMRT	44.4	19.5	31.0	43.2	19.7	31.2	2.0	0.1	6.2	1268.8
9	Proton	29.7	19.7	33.3	49.6	29.3	50.9	1.3	0.0	3.6	735.4
	IMRT	41.9	16.5	27.9	42.0	19.7	34.2	3.1	0.1	6.0	1222.3
10	Proton	32.9	22.9	32.7	49.2	31.1	42.9	55.6	3.5	5.9	837.0
	IMRT	49.4	23.6	33.7	49.7	24.4	33.7	47.8	3.0	8.8	1249.1

In left femoral head, *D*_*max*_ exceeded 50 Gy in one patient (#3) for both proton and IMRT plans, and in another patient (#8) only for the proton plan. The RTOG dose constraint of the penile bulb (*D*_*mean*_ ≤ 52.5 Gy) was not met in one patient (#1) for both plans and in another patient (#10) for the proton plan only

^a^In Gy

^b^Integral dose in Gycm^3^

## Abbreviations

GTV: Gross intraprostatic tumor volumes
IMRT: Intensity-modulated radiation therapy
PTV: Planning target volume
PPS: PTV of prostate
PGTV: PTV of gross tumor volume
PSV: PTV of seminal vesicles
CTV: Clinical target volume
PPS-PGTV: PPS subtracted by PGTV; PSV-PPS PSV subtracted by PPS
RTOG: Radiation Therapy Oncology Group
PD: Prescribed doses
*D*_*max*_: Maximum dose
*D*_*min*_: Minimum dose
*D*_*mean*_: Mean dose
SOBP: Spread-out Bragg peak
COG: Children’s Oncology Group
IC: Inhomogeneity coefficient
CN: Conformity number
